# Transcriptomic analysis of RDX and TNT interactive sublethal effects in the earthworm *Eisenia fetida*

**DOI:** 10.1186/1471-2164-9-S1-S15

**Published:** 2008-03-20

**Authors:** Ping Gong, Xin Guan, Laura S Inouye, Youping Deng, Mehdi Pirooznia, Edward J Perkins

**Affiliations:** 1 SpecPro Inc., 3909 Halls Ferry Road, Vicksburg, MS 39180, USA; 2 Environmental Laboratory, U.S. Army Engineer Research and Development Center, 3909 Halls Ferry Road, Vicksburg, MS 39180, USA; 3 Department of Biological Sciences, University of Southern Mississippi, Hattiesburg, MS 39406, USA; 4 Current address: Washington State Department of Ecology, P.O. Box 47600, Olympia, WA 98504, USA

## Abstract

**Background:**

Explosive compounds such as TNT and RDX are recalcitrant contaminants often found co-existing in the environment. In order to understand the joint effects of TNT and RDX on earthworms, an important ecological and bioindicator species at the molecular level, we sampled worms (*Eisenia fetida*) exposed singly or jointly to TNT (50 mg/kg soil) and RDX (30 mg/kg soil) for 28 days and profiled gene expression in an interwoven loop designed microarray experiment using a 4k-cDNA array. Lethality, growth and reproductive endpoints were measured.

**Results:**

Sublethal doses of TNT and RDX had no significant effects on the survival and growth of earthworms, but significantly reduced cocoon and juvenile counts. The mixture exhibited more pronounced reproductive toxicity than each single compound, suggesting an additive interaction between the two compounds. In comparison with the controls, we identified 321 differentially expressed transcripts in TNT treated worms, 32 in RDX treated worms, and only 6 in mixture treated worms. Of the 329 unique differentially expressed transcripts, 294 were affected only by TNT, 24 were common to both TNT and RDX treatments, and 3 were common to all treatments. The reduced effects on gene expression in the mixture exposure suggest that RDX might interact in an antagonistic manner with TNT at the gene expression level. The disagreement between gene expression and reproduction results may be attributed to sampling time, absence of known reproduction-related genes, and lack of functional information for many differentially expressed transcripts. A gene potentially related to reproduction (echinonectin) was significantly depressed in TNT or RDX exposed worms and may be linked to reduced fecundity.

**Conclusions:**

Sublethal doses of TNT and RDX affected many biological pathways from innate immune response to oogenesis, leading to reduced reproduction without affecting survival and growth. A complex interaction between mixtures of RDX and TNT was observed at the gene expression level that requires further study of the dynamics of gene expression and reproductive activities in *E. fetida*. These efforts will be essential to gain an understanding of the additive reproductive toxicity between RDX and TNT.

## Background

RDX and TNT are both important ordnance constituents often found coexisting in the environment [1,2]. A considerable number of studies have shown that TNT and RDX are highly toxic to soil invertebrates [3]. However, these two compounds appear to differ markedly in their modes of toxicological action. TNT is lethal to the earthworm *Eisenia fetida* with an LC_50_ of 120 mg/kg soil [3], whereas RDX reduces juvenile production (EC_50_ = 5 mg/kg soil [4]) without causing lethality at concentrations up to 756 mg/kg soil [5]. In contrast to TNT, which causes oxidative stress [6,7], RDX is known to act on the central nervous system causing seizures in humans and animals [8] and inducing neurotoxicological symptoms such as rigidity and ataxia in earthworms [9]. Sublethal doses of TNT affected the nervous system, caused blood disorders similar to methemoglobinemia, and weakened immunity in *E. fetida *[10]. However, the toxicological mechanisms of RDX as well as interactions between RDX and TNT are still largely unknown in earthworms.

Earthworms were described by Aristotle as “the intestines of the earth” and have been used as bioindicators for soil contamination [11]. In the present study, we investigated the sublethal transcriptional response in *E. fetida* exposed to a mixture of RDX and TNT in comparison to worms exposed to TNT or RDX alone. We performed earthworm reproductive toxicity tests and measured gene expression in exposed and unexposed worms. We hypothesized that worms exposed to RDX, TNT or a mixture of these two compounds would show toxicant-specific gene expression profiles. Our objectives were to (1) identify earthworm genes affected by TNT and RDX singly or in combination; (2) examine the interactive effects between TNT and RDX by comparing their gene expression profiles; and (3) gain some mechanistic insights into the toxicological modes of action for exposures to mixtures of TNT and RDX.

## Results

Adult worms were exposed for 28 d to 50 mg TNT/kg soil, 30 mg RDX/kg soil, or both. The TNT or RDX concentration was each selected to target an EC_50_ of the cocoon production endpoint (L.S. Inouye, unpublished data). Our results of cocoon counts are consistent with the target value for these two compounds (Table 1). We observed no statistically significant effects on mortality and growth. Reproduction endpoints were determined at 56 d and significant decreases in cocoon/juvenile counts were recorded in treated samples. The mixture of TNT and RDX inhibited offspring production more than TNT or RDX alone did (Table 1).

**Table 1:** Results of the 56-d earthworm reproductive toxicity test. Data are given in mean ± standard error (*n* = 20 jars, 5 adult worms added in each jar) followed by a letter indicating significant (if different, *p* < 0.05) or insignificant difference (if the same, *p* > 0.05) from the control (ANOVA). The nominal concentrations of TNT and RDX are 50 mg/kg soil and 30 mg/kg soil, respectively.

Treatment	Adult survival (%)	Growth in weight loss (mg)	Cocoon counts (# per jar)	Juvenile counts (# per jar)
**Control**	100 ± 0 a	10 ± 4 a	9.5 ± 1.1 a	15.7 ± 2.6 a
**TNT**	96 ± 4 a	126 ± 110 a	5.1 ± 1.0 b	1.4 ± 1.0 b
**RDX**	98 ± 1 a	7 ± 8 a	5.2 ± 0.7 b	2.4 ± 1.0 b
**TNT + RDX**	95 ± 3 a	99 ± 82 a	0.8 ± 0.2 c	0.6 ± 0.5 b

**Figure 1:**
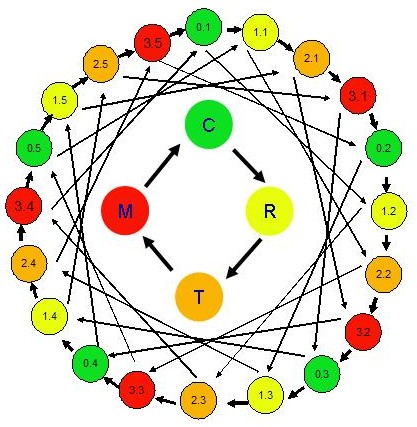
A balanced loop hybridization scheme for four treatments with five independent biological replicates. Circles represent treatment samples. Sample code: 0.x = replicate x of solvent control worms (C); 1.x = replicate x of 30 mg RDX/kg soil treated worms (R); 2.x = replicate x of 50 mg TNT/kg soil treated worms (T); 3.x = replicate x of 50 mg TNT and 30 mg RDX/kg soil treated worms (M); x = 1-5. Arrows represent array hybridizations between respective samples where the arrowhead indicates Alexa 647 dye labeling and the base of arrows indicate Cy3 dye labeling. See Additional file Additional file 1: Table S1 for more details of the hybridization scheme.

Gene expression was examined in five biological replicates per treatment, that is, one worm from each of the five replicate samples per treatment using 40 earthworm cDNA microarrays and a balanced interwoven loop design [12] (Figure 1 and Additional file Additional file 1: Table S1). Two mixtures of Stratagene Alien® mRNAs were spiked into the worm mRNA samples (see Methods and Table 2) for quality assurance of cDNA synthesis, labeling and hybridization as well as for monitoring the procedure and power of statistical analysis in deriving differentially expressed genes. All the 8704 features on the array were treated as individual genes although each cDNA and control spots were duplicated on the array. We adjusted the confidence level of the false discovery rate and the maximum allowable false discovery genes within the two-class comparison algorithm implemented in BRB Array Tools (see Methods) so that the Alien® mRNAs that were spiked in two different treatments (e.g., control vs. RDX and control vs. mixture) at a ratio of 2 or 0.5 would consistently show up on the significant gene list, while those at a ratio of 1 would not (Table 2). Under the selected conditions (95% confidence level and 10 allowable false discoveries), we identified 151 and 96 significant genes in the RDX- and mixture-treated worms, respectively, without any alien spike 3 (Table 2 and Additional file Additional file 2: Table S2), suggesting no false positive from the spike-in mRNAs. In the two treatments added with the same Alien® mRNA spike-in mix (i.e., control vs. TNT), we identified 957 significant genes including 5 spots of alien spike 3 and 9 spots of negative controls (water and printing buffer), which is reasonable given the large number of significant genes called and the selected inference conditions. Therefore, the use of the spike-in RNAs allowed us to identify at least two-fold expression difference between treatments in our dataset at an acceptable false discovery rate. This practice also validates the statistical program used for array data analysis and gives us a higher confidence in the inferred significant genes.

**Table 2:** Composition of Alien® mRNA spike-in mix, 0.5 µl of which was used in every 6-µl cDNA synthesis reaction. Mix1 was added to the Control and the TNT treatment group while Mix2 to the RDX and TNT+RDX groups.

**Alien spike**	**Mix1 (ng/µl)**	**Mix2 (ng/µl)**	**Ratio Mix1/2**	**Ratio Mix2/1**
AS1	0.1	0.05	2	0.5
AS2	0.05	0.1	0.5	2
AS3	0.01	0.01	1	1
AS4	0.005	0.001	5	0.2
AS5	0.001	0.005	0.2	5

**Figure 2:**
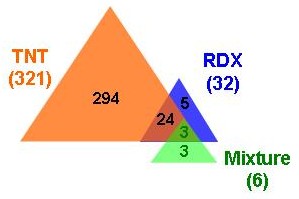
The numbers and overlapping of differentially expressed genes in all three treatments as compared with the controls that are inferred using BRB Array Tools.

**Figure 3:**
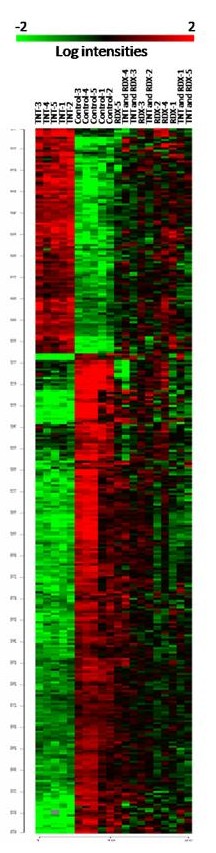
Expression heat map of 329 differentially expressed transcripts in earthworms exposed to TNT, RDX or TNT+RDX in comparison with the controls (five worms per treatment). Both samples and genes were hierarchically clustered using Euclidean distance and average linkage (see Additional file Additional file 4: Figure S1 and Additional file Additional file 5: Figure S2 for dendrograms).

**Figure 4:**
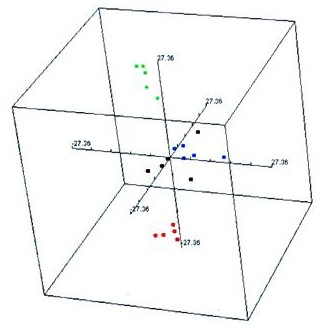
Multidimensional scaling analysis of the 20 earthworm mRNA samples using Euclidean distance and the expression dataset for the 329 significant genes. Color coding for samples: green = control treatment, red = TNT, black = RDX, and blue = mixture.

To further increase the inference stringency and to down-select a smaller number of genes for future experimental validation, we applied the following criterion: the expression of a gene must show statistically significant difference between the treated and the control at both duplicate spots to be inferred as a differentially expressed gene. After applying this criterion, we identified 321 significant differentially expressed genes in TNT treated worms, 32 in RDX treated worms, and only 6 in mixture treated worms in comparison with the controls (two spots account for one gene) (Figure 2). Three genes were significantly altered by all three treatments, 24 genes were common between TNT treated and RDX treated worms, and 302 genes were unique to the three treatments. The expression of all 329 inferred significant genes is shown in Figure 3 and is also given in Additional file Additional file 3: Table S3(a) along with their annotation information. Both multidimensional scaling analysis (equivalent to principal component analysis; Figure 4) and hierarchical clustering (Additional file Additional file 4: Figure S1 and Additional file Additional file 5 figure S2) of the 20 earthworm mRNA samples using the significant gene set indicate the greatest distance between the TNT treatment and the control treatment. Samples from the control and TNT treatments form two well separated clusters with samples from the RDX and mixture treatments forming a third unresolved cluster (Figure 4). These results suggest that gene expression profiles of TNT treated worms are most distinct from those of the controls and that those of RDX and mixture treated worms cannot be separated. The differences in gene expression profile between the three explosive treatments largely come from the degree of alteration of the 329 significant genes as evidenced by the high correlation in both absolute and relative mean expression (Table 3). Only in very few occasions (9/658 or 1.4%), genes altered by TNT exposure showed an opposite direction of regulation by the RDX or mixture treatment. However, none of these cases reached a degree of statistical significance and 7 out of 9 cases occurred in one of the duplicated spots (Additional file Additional file 3: Table S3(a)).

**Table 3:** Correlation coefficient of the mean expression of 329 significant genes between different treatments based (*n* = 2×329) (see Additional file Additional file 3: Table S3(a) for expression data).

	Geometric mean of expression				Expression relative to the control		
Treatment	Control	RDX	TNT	Mixture	RDX	TNT	Mixture
Control		0.602	0.232	0.655			
RDX			0.836	0.953		0.908	0.861
TNT				0.785			0.908
Mixture							

## Discussion

We profiled gene expression in earthworms exposed to TNT (50 mg/kg soil), RDX (30 mg/kg soil) or a mixture of these two explosives using a 4K cDNA microarray. Consistent with our earlier study [10], we identified genes related to multiple pathways being affected by TNT: genes involved in oxygen transport and iron homeostasis (e.g., ferritin), blood coagulation and fibrinolysis (fibrinogen and fibronectin), muscle contraction and cell motility (actin, tropomyosin and troponins), immune response (chitinase and peptidoglycan recognition protein), antioxidant response (metallothionein, glutathione S-transferase, cytochrome c oxidase, and NADH dehydrogenase), calcium signaling (centrin and other proteins containing calcium binding EGF-like domains), protein degradation (lysozyme destabilase and ubiquitylation). We also observed differential expression of quite a few transcripts which were not detected previously. Putatively, these transcripts are involved in not only some previously identified pathways (e.g., proteins containing spectrin repeats or actin binding domains and a Kelch-like protein, all involved in cell motility), but also many other important pathways not previously identified (Additional file Additional file 3: Table S3(a)). These newly identified pathways include ribosomal structure, translation, posttranslational modification, protein turnover, protein transport, non-lysosomal protein degradation, protein-protein interaction, protease inhibitors, energy homeostasis, glycolysis, and many signal transduction pathways (e.g., protein phosphorylation and ADP ribosylation). In support of our previous findings of adverse neurological effects, we observed significant changes in the expression of several genes involved in Notch and agrin signaling pathways which block neuronal differentiation [13] and regulate acetylcholine receptors in neuromuscular junctions [14], respectively.

All chemical treatments caused significant reduction in reproductive output. Hence transcripts related to reproductive functions are of considerable interest in order to link effects on the gene expression level to phenotypic effects. Among the 329 significant genes, there is one transcript putatively coding for echinonectin involved in oogenesis. Echinonectin, a dimeric galactosyl-binding protein found deposited to the inner side of the sea urchin egg's hyaline layer (extracellular matrix) [15,16] may be related to cocoon laying processes in earthworms. Down-regulation of this transcript may contribute to the reduced cocoon and juvenile counts in treated worms.

There is a novel differentially expressed transcript similar to myeloid differentiation factor 88 (*E* = 2×10^-13^) containing a Toll/interleukin-1 resistance (TIR) domain (Additional file Additional file 3: Table S3(a)). Presence of this transcript suggests that earthworms may possess components of Toll or Toll-like receptor signaling pathways in contrast to current views [17,18]. It has been shown that the TIR domain is required for *Caenorhabditis elegans* resistance to microbial pathogens [19]. Our data suggests that this gene was down-regulated in all treated worms (Additional file Additional file 3: Table S3(a)), implying that exposures to explosives had weakened the innate immune system in *E. fetida*, consistent with our earlier findings [10]. Further work is currently underway to identify other Toll-related genes and establish this highly conserved pathway in *E. fetida*.

Surprisingly, the microarray data indicate that TNT exhibited the greatest effect on earthworm gene expression with the vast majority of the 329 significant genes significantly changed by TNT but not the mixture of TNT and RDX (Figure 3 and Additional file Additional file 3: Table S3(a)). The greater impact of TNT on gene expression as opposed to the mixture is the reverse of the additive effect seen in reproductive toxicity results where the mixture has a far greater effect (Table 1). While TNT exposures dominated expression effects, the mixture did have a greater impact on a few genes whose expression was not significantly affected by TNT or RDX (Additional file Additional file 3: Table S3(a)). Furthermore, the mean values for expression of several other genes (Additional file Additional file 3: Table S3(b) and Additional file Additional file 6: Table S4) are correlated with cocoon production and hence appear to reflect an additive effect. For instance, a two-tailed *t*-test suggests that the expression of transcripts putatively coding for ferritin heavy chain polypeptide 1, a chitinase, and dopamine β-monooxygenase was more depressed in the mixture-treated worms than in TNT or RDX-treated worms (Additional file Additional file 3: Table S3(b)). The highly conserved gene ferritin plays an important role in gametogenesis, fertilization, or early embryonic development [20]. While the mean expression of ferritin, chitinase, and dopamine β-monooxygenase was correlated to the additive toxicity effect we have observed (Table 1 and Additional file Additional file 3: Table S3(b)), we are unable to identify genes that can mechanistically account for the apparent additive effect between TNT and RDX on reproduction. This may be in part due to a failure to capture gene expression changes leading to critical reproductive events that may have occurred prior to the time when the worms were sampled. In particular, earthworms are hermaphroditic (i.e., each individual possesses both male and female organs), and their reproductive activities are complex and involve sequentially mating, sperm exchange, egg fertilization and cocoon deposition. Failure to capture these events may also be due to the nature of the cDNA array because the cDNA libraries used cover only a fraction of the *E. fetida* genome [21]. Genes related to reproductive activities such as fertilization in earthworms (except echinonectin) may be underrepresented on the microarray, as is exemplified by the apparent absence from the array of annetocin, a gene involved in the induction of egg-laying behavior through its action on the nephridia in *E. fetida*[22,23]. A significant problem with earthworm libraries, a non-model organism, is that many cDNAs remain unidentified. Among the 329 significant genes, 68% have poor (*E* > 10^-5^) or no blastx matches. The poorly characterized transcripts constitute two-thirds of the total unique sequences in our libraries [21], a major obstacle in linking gene expression to toxicological endpoints with ecological or physiological relevance (e.g., reproduction). We are now making more efforts toward transcriptome-wide sequencing and annotation in *E. fetida*.

## Conclusions

Both toxicity and gene expression results indicate that TNT and RDX, singly or jointly cause adverse effects in *E. fetida*. We identified transcripts putatively coding for echinonectin, ferritin heavy chain polypeptide 1, chitinase and dopamine β-monooxygenase that can link reproductive toxicological endpoints with gene expression. Although the mixture of TNT and RDX caused an additive impact on reproductive endpoints, expression of the 329 inferred significant transcripts was affected more by TNT than by RDX or the mixture. RDX showed an antagonistic effect with TNT on the expression of the majority of the 329 differentially expressed transcripts. We also identified a novel gene involved in Toll signaling pathway that was previously thought non-existent in the Oligochaete earthworms. However, only a relatively small number of well-characterized earthworm genes have been spotted on our microarray. More efforts in sequencing, annotation and construction of gene regulatory networks are needed to better understand the interactions between genes involved in different biological processes or pathways. Gene expression in earthworms should also be profiled at the specific and well-defined physiological states to catch the genes relevant to the assayed toxicological endpoints.

## Methods

### Animal culture and chemicals

*E. fetida* were maintained in a continuous culture from stocks obtained from Carolina Biological Supply Company (Burlington, NC, USA). Worms were kept at 22 to 25°C in moistened sphagnum peat with calcium carbonate added to adjust the pH to 6.5 to 7.5 and moisture content adjusted to 50% and were fed ad libitum on a diet of Magic Worm Food (Carolina Biological Supply). Fully clitellate adults weighing 0.3-0.6 g (live weight) were selected for all experiments. TNT (CAS no. 118-96-7, purity > 99%) was purchased from Chem Service (West Chester, PA, USA). RDX (purity > 99%) was obtained from Stan Caulder (Naval Surface Warfare Center, Indianhead, MD, USA).

### Printing of cDNA microarrays

Two cDNA libraries with a total of 4032 clones were constructed using the suppression subtractive hybridization technique [24] from earthworms of the same continuous culture as was used for TNT and/or RDX exposures and analysis, details of which can be found in a separate publication [21]. Two microliters of each clone culture was amplified in a 100-µl PCR reaction, followed by PCR product purification using Millipore Montage PCR 96 Cleanup Kit. We randomly checked the concentration of purified cDNA using PicoGreen dsDNA Quantitation Kit (Molecular Probes, Eugene, OR, USA) and found it ranged 100~500 ng/µl with an average of 240 ng/µl. All the purified cDNA amplicons were loaded on 384-well plates and dried completely in a Vacufuge^TM^ Concentrator 5301 (Eppendorf, Westbury, NY, USA). The dried cDNAs were re-suspended in 15 µl of 1× printing buffer (ArrayIt, Sunnyvale, CA, USA). Each clone was spotted once in each of two super grids on Ultra GAPS^TM^ amino silane coated glass slides (Corning, Acton, MA, USA) using 16 pins on a VersArray ChipWriter (Bio-Rad, Hercules, CA, USA). Five spike-in control cDNAs, i.e., PCR product 1 to 5 selected from SpotReport® Alien® cDNA Array Validation System (Stratagene, La Jolla, CA, USA) prepared at 15, 30, 60, 125 and 250 ng/µl, were also spotted twice along with printing buffer and water as control spots. After printing, arrays were incubated in a dessicator 2-3 days and were then snap-dried on a hot plate after being rehydrated over a boiling water bath. The arrays were further immobilized using a UV Cross-linker (Stratagene) by applying 300 mJ per 10 arrays. The annotated earthworm cDNA microarray v. 1.1 (GEO platform accession number GPL5667) contained 8704 features including 60 alien cDNA spots, 84 water spots, 256 blank spots and 240 printing buffer spots.

### Earthworm toxicity test

Standard 56-d reproductive toxicity tests were conducted in a field collected silty loam soil (3% sand, 72% silt, 26% clay, pH 6.7, total organic C 0.7%, and CEC 10.8 mEq/100 g [25]) in an environmental chamber with continuous lighting and temperature maintained at 21±1°C in accordance with the ASTM guideline [26]. Appropriate amounts of TNT and/or RDX dissolved in acetone were spiked into air-dried soil to achieve the following nominal concentrations: 0 (solvent control), 50 mg TNT/kg soil 30 mg RDX/kg soil, and 50 mg TNT + 30 mg RDX/kg soil. Acetone was allowed to evaporate 2 d before rehydrating the soil to 85% of its water-holding capacity (i.e., 0.295 ml/g). The rehydrated soils were allowed to equilibrate for 7 d before being used for earthworm toxicity testing. Five mature worms were added in a jar containing 250 g (dry weight equivalent) of TNT/RDX amended or non-amended soil. Each treatment had 20 replicate jars. Adult worms were removed, counted, and weighed at 28-d with one worm from each of 10 replicate jars per treatment reserved for gene expression in an RNAlater-ICE solution (Ambion, Austin, TX, USA) at -80°C and remaining worms snap-frozen at -80°C for other future uses. Juveniles and cocoons (both hatched and unhatched) were counted in the remaining 10 replicate jars per treatment at 56-d.

### Hybridization probe preparation

Total RNA was isolated using Qiagen RNeasy Mini Kit (Valencia, CA, USA) from five of the 10 RNAlater-ICE preserved worm tissues per treatment (each worm was chopped into 8~10 pieces) and was pooled as one biological replicate representing each individual earthworm. The pooled total RNA was purified to obtain mRNA using NucleoTrap Nucleic Acid Purification Kit (BD Biosciences, Franklin Lakes, NJ, USA). Nuclease-free water (Ambion) was used to elute both total and poly(A) mRNA. RNA concentration and quality were measured using NanoDrop® ND-1000 Spectrophotometer (NanoDrop Technologies, Wilmington, DE, USA). Worm mRNA (30 ng, A260/A280 ratio = 1.9~2.1) together with Stratagene SpotReport® Alien® spike-in mRNAs corresponding to the SpotReport® Alien® cDNAs spotted on the array was reverse-transcribed to cDNA using SuperScript^™^ III reverse transcriptase (Invitrogen, Carlsbad, CA, USA) and RT primer included in the Genisphere 3DNA 900 Expression Array Detection kit (Hatfield, PA, USA).

### Hybridization and array scanning

Each biological replicate of cDNA samples was hybridized four times on four different arrays with two swaps of Cy3 and A647 fluorescence dyes. Prior to hybridization spotted cDNA arrays were pre-washed in 5× SSC, 0.1% SDS and 0.1 mg/ml BSA for 45-60 min at 42°C, then immersed in 0.1× SSC for 2×5 min and in RNase-free water for 30 s at room temperature, and dried by centrifugation at 2500 rpm for 2 min. Synthesized cDNA probes were labeled and hybridized to the array using Genisphere 3DNA 900 Expression Array Detection Cyanine 3 or Alex Fluor 647 Kit by following manufacture's protocol. After hybridization, arrays were scanned at 5-µm resolution using a VersArray ChipReader (Bio-Rad). Raw spot and background signal intensities (mean and standard deviation) were acquired by processing scanned array images on VersArray Analyzer Software version 4.5 (Bio-Rad).

### Microarray data analysis

A spot was flagged if it was oversaturated or its raw signal intensity was below its background level or the mean signal intensity plus 2× standard deviation of the 256 blank spots on either channel. The filtered data was normalized by background subtraction and centering to each channel's mean spot intensity. The raw and processed signal data of all 40 arrays have been deposited in the GEO database with a series number of GSE8909. Statistical two-class comparison between the control and the treated groups was performed using the BRB Array Tools version 3.6.0 beta_2 release (). Prior to the analysis, the geometric mean of processed four technical replicates (i.e., four hybridization with twice Cy3-labeled and twice A647-labeled) for each biological sample was collated as a single-channel dataset (Additional file Additional file 6: Table S4) and features were excluded if more than 50% of data was filtered out. The remaining 7708 features were analyzed at the following setting: two-sample T-test with random variance model and 126 available multivariate permutations, 95% confidence level of false discovery rate assessment, and 10 maximum allowable false-positive genes.

## Abbreviations

RDX – acronym for Royal Demolition Explosive with an IUPAC name of 1,3,5-trinitro-1,3,5-triazacyclohexane;

TNT – 2,4,6-Trinitrotoluene;

LC_50_ – A lethal concentration at which 50% of the exposed organisms are dead;

EC_50_ – An effective concentration at which 50% of the exposed organisms are affected;

## Competing interests

The authors declare that they have no competing interests.

## Authors' contributions

PG, LSI and EJP designed and coordinated the study. LSI performed the reproductive toxicity tests. XG and PG printed the arrays, extracted worm mRNA and conducted array hybridization. YD and MP provided gene annotation. PG analyzed and submitted the array data to the GEO database and drafted the manuscript. PG and EJP revised and finalized the manuscript which was read and approved by all authors.

## Supplementary Material

Additional file 1 Table S1. Hybridization scheme and array data deposition information. Click here for file

Additional file 2 Table S2. The original significant gene lists derived using BRB Array Tools. Click here for file

Additional file 3 Table S3. (a) Significant genes inferred from the microarray experimental data with expression and annotation information; (b) Average gene expression affected by mixture more than by TNT or RDX: examples. Click here for file

Additional file 4 Figure S1. Dendrogram for clustering earthworm mRNA samples using Euclidean distance and average linkage. Click here for file

Additional file 5 Figure S2. Dendrogram for clustering 329 significant earthworm transcripts using Euclidean distance and average linkage. Click here for file

Additional file 6 Table S4. The collated array dataset for statistical analysis. Click here for file
