# Supplementary material for: Transcriptomic analysis of RDX and TNT interactive sublethal effects in the earthworm Eisenia fetida
Source: BMC Genomics. 2008 Mar 20;9(Suppl 1):S15. doi: 10.1186/1471-2164-9-S1-S15 (PMC2386057; doi:10.1186/1471-2164-9-S1-S15)
Supplement: Additional file 1 — Table S1. Hybridization scheme and array data deposition information. [file 1471-2164-9-S1-S15-S1.pdf]

Table S1. Hybridization scheme and array data deposition information. The complete dataset of 40 arrays/hybridizations was deposited in GEO with a series accession number of GSE8909 and a platform accession number of GPL5776.

Hybridization	Cy3	A647	Slide barcode	GEO accession #
1	TNT+RDX-5	Control-1	13468230	GSM225617
2	Control-1	RDX-1	13468227	GSM225602
3	TNT+RDX-1	Control-2	13468226	GSM225597
4	Control-2	RDX-2	13468229	GSM225605
5	TNT+RDX-2	Control-3	13468225	GSM225592
6	Control-3	RDX-3	13468224	GSM225588
7	TNT+RDX-3	Control-4	13468221	GSM225570
8	Control-4	RDX-4	13468222	GSM225581
9	TNT+RDX-4	Control-5	13468219	GSM225550
10	Control-5	RDX-5	13468233	GSM225621
11	Control-1	RDX-2	13468235	GSM225624
12	TNT+RDX-1	Control-3	13468237	GSM225625
13	Control-2	RDX-3	13468240	GSM225628
14	TNT+RDX-2	Control-4	13468241	GSM225629
15	Control-3	RDX-4	13468239	GSM225627
16	TNT+RDX-3	Control-5	13468238	GSM225626
17	Control-4	RDX-5	13468242	GSM225638
18	TNT+RDX-4	Control-1	13468245	GSM225643
19	Control-5	RDX-1	13468244	GSM225640
20	TNT+RDX-5	Control-2	13468243	GSM225639
21	RDX-1	TNT-1	13436264	GSM225531
22	TNT-1	TNT+RDX-1	13436254	GSM225352
23	RDX-2	TNT-2	13436250	GSM225345
24	TNT-2	TNT+RDX-2	13436152	GSM225341
25	RDX-3	TNT-3	13436255	GSM225359
26	TNT-3	TNT+RDX-3	13436237	GSM225343
27	RDX-4	TNT-4	13436249	GSM225344
28	TNT-4	TNT+RDX-4	13436150	GSM225340
29	RDX-5	TNT-5	13436148	GSM225315
30	TNT-5	TNT+RDX-5	13436153	GSM225342
31	RDX-1	TNT-2	13436253	GSM225346
32	TNT-1	TNT+RDX-2	13468777	GSM225646
33	RDX-2	TNT-3	13468775	GSM225644
34	TNT-2	TNT+RDX-3	13468232	GSM225620
35	RDX-3	TNT-4	13468778	GSM225650
36	TNT-3	TNT+RDX-4	13436149	GSM225321
37	RDX-4	TNT-5	13436258	GSM225525
38	TNT-4	TNT+RDX-5	13468220	GSM225559
39	RDX-5	TNT-1	13468215	GSM225536
40	TNT-5	TNT+RDX-1	13468234	GSM225623
