# Supplementary material for: Transcriptomic analysis of RDX and TNT interactive sublethal effects in the earthworm Eisenia fetida
Source: BMC Genomics. 2008 Mar 20;9(Suppl 1):S15. doi: 10.1186/1471-2164-9-S1-S15 (PMC2386057; doi:10.1186/1471-2164-9-S1-S15)
Supplement: Additional file 4 — Figure S1. Dendrogram for clustering earthworm mRNA samples using Euclidean distance and average linkage. [file 1471-2164-9-S1-S15-S4.pdf]

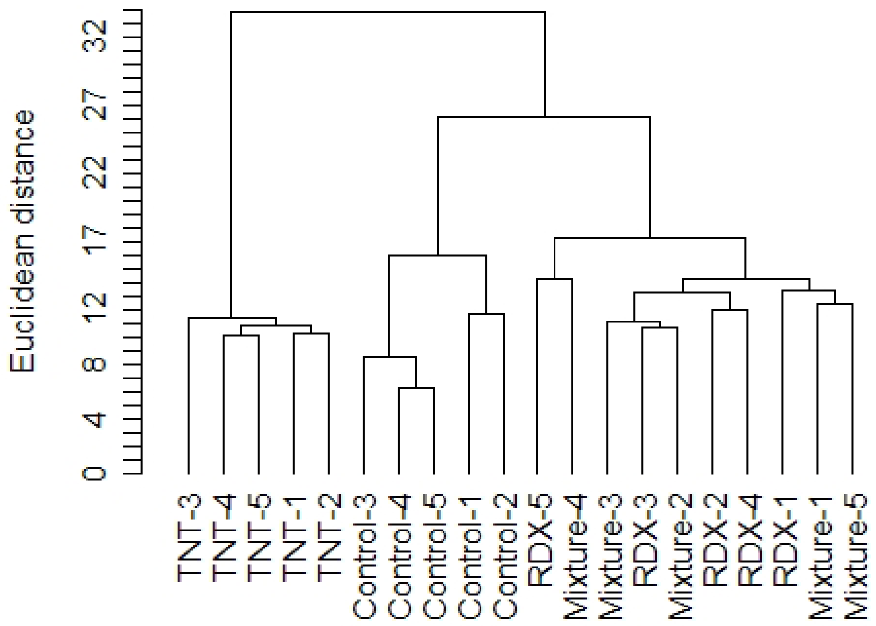

Figure S1. Dendrogram for clustering earthworm mRNA samples using Euclidean distance and average linkage.
