# Supplementary material for: Transcriptomic analysis of RDX and TNT interactive sublethal effects in the earthworm Eisenia fetida
Source: BMC Genomics. 2008 Mar 20;9(Suppl 1):S15. doi: 10.1186/1471-2164-9-S1-S15 (PMC2386057; doi:10.1186/1471-2164-9-S1-S15)
Supplement: Additional file 5 — Figure S2. Dendrogram for clustering 329 significant earthworm transcripts using Euclidean distance and average linkage. [file 1471-2164-9-S1-S15-S5.pdf]

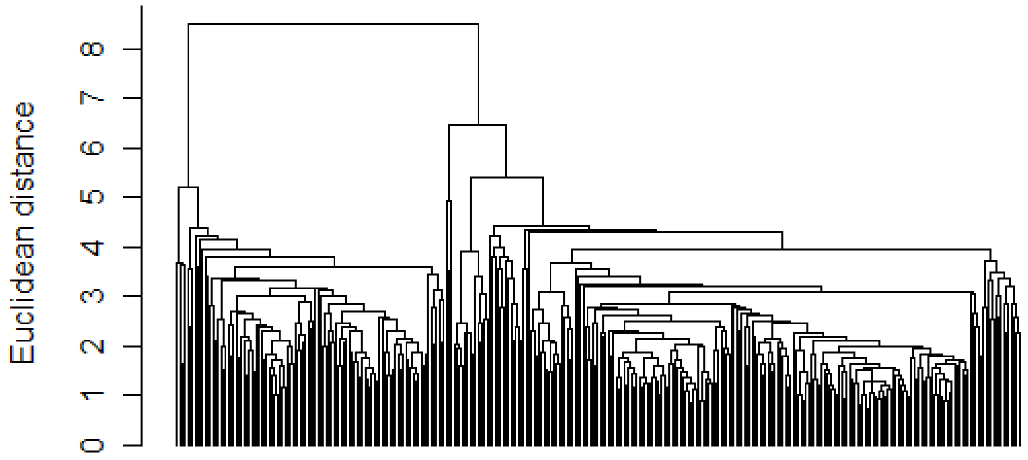

Figure S2. Dendrogram for clustering 329 significant earthworm transcripts using Euclidean distance and average linkage.
